# Novel Lyssavirus in Natterer’s Bat, Germany

**DOI:** 10.3201/eid1708.110201

**Published:** 2011-08

**Authors:** Conrad M. Freuling, Martin Beer, Franz J. Conraths, Stefan Finke, Bernd Hoffmann, Barbara Keller, Jeannette Kliemt, Thomas C. Mettenleiter, Elke Mühlbach, Jens P. Teifke, Peter Wohlsein, Thomas Müller

**Affiliations:** Author affiliations: Friedrich-Loeffler-Institut, Wusterhausen, Germany (C.M. Freuling, F.J. Conraths, J. Kliemt, T. Müller);; Friedrich-Loeffler-Institut, Greifswald-Insel Riems, Germany (M. Beer, S. Finke, B. Hoffmann, T.C. Mettenleiter, J.P. Teifke);; Lower Saxony State Office for Consumer Protection and Food Safety, Hannover, Germany (B. Keller);; Nature and Biodiversity Conservation Union, Berlin, Germany (E. Mühlbach);; University of Veterinary Medicine, Hannover (P. Wohlsein)

**Keywords:** rabies, bats, lyssavirus, Natterer’s bat, Myotis nattereri, sequence analysis, zoonosis, Germany, viruses, dispatch

## Abstract

A virus isolated from a Natterer’s bat (*Myotis nattererii*) in Germany was differentiated from other lyssaviruses on the basis of the reaction pattern of a panel of monoclonal antibodies. Phylogenetic analysis supported the assumption that the isolated virus, Bokeloh bat lyssavirus, may represent a new member of the genus *Lyssavirus*.

Bats have been identified as carriers or reservoirs for a plethora of viruses, including human pathogens like severe acute respiratory syndrome coronavirus, henipaviruses, filoviruses, or lyssaviruses, which cause rabies (1). The genus *Lyssavirus* within the family *Rhabdoviridae* contains 11 viruses: rabies virus (RABV), Lagos bat virus, Mokola virus, Duvenhage virus, European bat lyssaviruses types 1 and 2 (EBLV-1 and EBLV-2), Australian bat lyssavirus, Aravan virus (ARAV), Khujand virus (KHUV), Irkut virus, and West Caucasian bat virus (2). A proposed new species, Shimoni bat virus, has recently been isolated from *Hipposideros commersoni* leaf-nosed bats (3). Although RABV, which circulates in dogs, causes most of the ≈55,000 human deaths from rabies per year, most bat lyssaviruses have been demonstrated to cause human rabies (4).

From 1977 through 2009, a total of 928 cases of bat rabies (EBLV-1 and EBLV-2) were detected in Europe, but only 10 of the 45 known indigenous bat species tested positive for lyssavirus; most were serotine bats (*Eptesicus serotinus*) associated with EBLV-1 (5,6). In Germany, bat rabies has been known since the middle of the 20th century, and most isolated viruses were characterized as EBLV-1 (6). EBLV-2 is associated with *Myotis* spp. bats (*M. daubentonii* and *M. dascyneme*) and has only sporadically been found in Europe and in Germany (7). The transmission of EBLV-1 and EBLV-2 in bats in Europe is still only poorly understood (8). We report lyssavirus infection in a Natterer’s bat.

## The Study

In November 2009, a bat was found on the ground in Bokeloh, Lower Saxony, Germany (52°25′13.99″N; 9°23′31.56″E). The bat was morphologically identified as a Natterer’s bat (*M. nattererii*). It was given mealworms and water ad libitum supplemented with minerals and vitamins. In February 2010, the bat began to act aggressively, directly approaching any moving object, vigorously trying to bite, and screaming ferociously. This agitated stage lasted for 7 days and was followed by general weakness, lethargy, and paralysis. After the first 3 days of the clinical course, the bat stopped drinking and eating. Ten days after recognition of the first clinical signs, the animal died.

**Figure 1:**
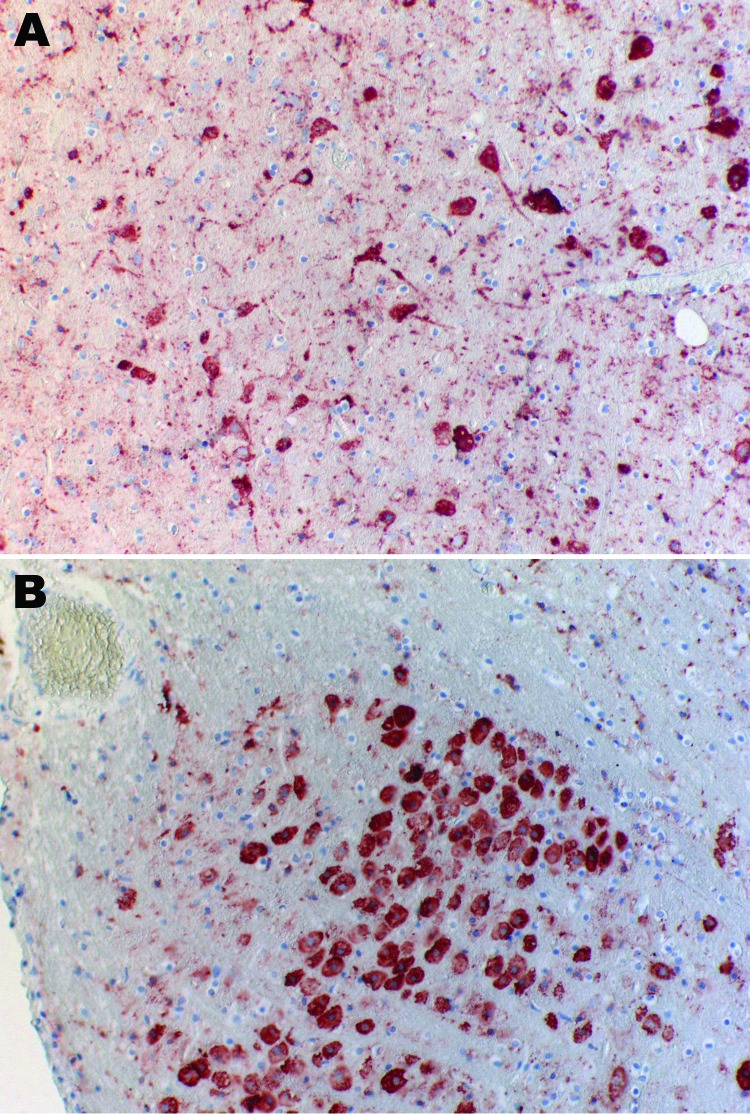
Immunohistochemical analysis of brain of Natterer’s bat for lyssavirus antigen by using the avidin biotin complex method. A) Cerebrum showing a large number of neurons. Cytoplasmic granular-to-diffuse staining for rabies antigen is visible in the perikarya and neuronal processus. B) Medulla and neurons of the nucleus funiculi lateralis showing strong cytoplasmic staining for rabies antigen. Original magnifications ×20.

The bat was submitted for testing, and rabies diagnosis was performed by using immunohistochemical analysis. Lyssavirus antigen was detected in numerous neurons of the cerebral cortex, cerebellum, and especially the nucleus funiculi lateralis and the nucleus olivaris of the medulla (Figure 1). Organs other than the central nervous system, e.g., the salivary glands, did not contain lyssavirus antigen. After the first cell passage in rabies tissue culture infection test (9), virus was isolated from brain tissue. Antigenic typing performed with a panel of 10 antinucleocapsid monoclonal antibodies (10) clearly differentiated the isolated virus from all other tested lyssavirus species (Table). Thus, the virus isolate was tentatively named Bokeloh bat lyssavirus (BBLV).

**Table:** Reactivity of 10 monoclonal antibodies against nucleocapsid protein of BBLV compared with 7 other lyssaviruses, Germany*

Antibody	RABV	LBV	MOKV	DUVV	EBLV-1	EBLV-2	ABLV	BBLV
W239.17	+++	+++	+++	+++	+++	+++	+++	+++
W187.5	+++	–	–	–	–	–	+++	–
W187.11.2	+++	–	–	–	–	–	+++	+++
MW187.6.1	+++	+++	+++	+++	–	–	+++	+++
MSA6.3	–	–	+++	–	+++	+++	–	+++
LBV7.36	–	+++	–	–	–	+++	–	–
DUV6.15.19	–	–	–	+++	+++	–	–	–
S62.1.2	–	–	–	–	+++	+++	–	–
P 41	–	–	–	–	–	–	–	–
Z144.88	–	–	–	–	–	–	–	–

*BBLV, Bokeloh bat lyssavirus; RABV, rabies virus; LBV, Lagos bat virus; MOKV, Mokola virus; DUVV, Duvenhage virus; EBLV, European bat lyssavirus; ABLV, Australian bat lyssavirus; +++, strongly positive; –, negative.

**Figure 2:**
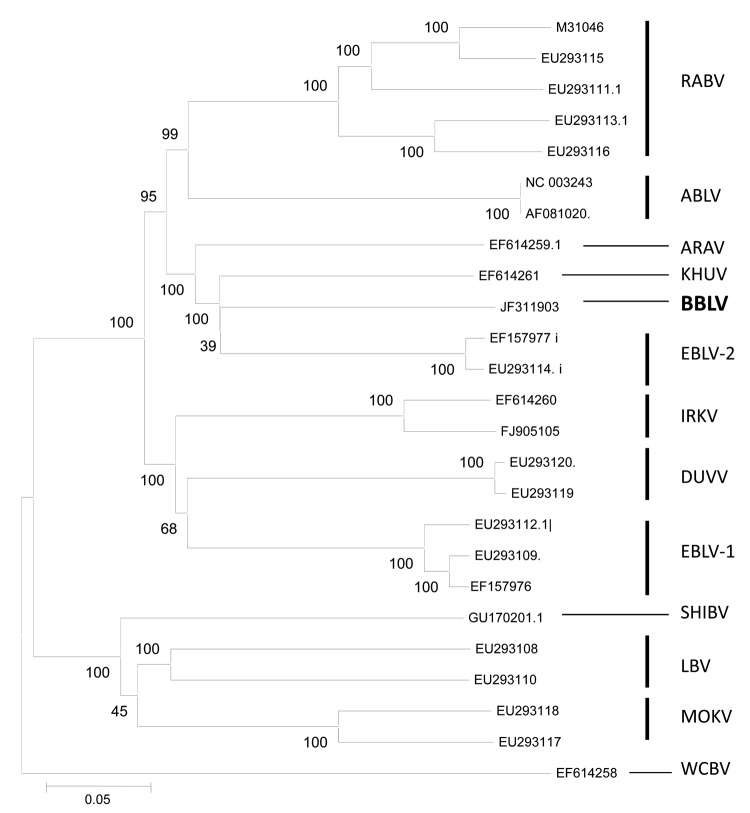
Phylogenetic tree inferred from concatenated N-P-M-G-L sequences of bat lyssaviruses. The neighbor-joining method (Kimura 2-parameter) was used as implemented in MEGA4 software (www.megasoftware.net). Bootstrap values (500 replicates) are shown next to branches. Scale bar indicates nucleotide substitutions per site. Virus isolated in this study is shown in **boldface**. RABV, rabies virus; ABLV, Australian bat lyssavirus; ARAV, Aravan virus; KHUV, Khujand virus; BBLV, Bokeloh bat lyssavirus; European bat lyssavirus; IRKV, Irkut virus; DUVV, Duvenhage virus; SHIBV, Shimoni bat virus; LBV, Lagos bat virus; MOKV, Mokola virus; WCBV, West Caucasian bat virus.

Results of discriminatory reverse transcription PCR results for EBLV-1 and EBLV-2 (11,12) were negative, and only a generic reverse transcription PCR (13) yielded a 605-bp amplification product similar to that of the positive control. The nucleotide sequence was determined by using standard methods (primers and protocols are available upon request). Sequence analysis of the nucleoprotein gene performed with MEGA version 4.0 software (www.megasoftware.net/mega4/mega.html) showed that BBLV differed from all other published lyssavirus sequences with the highest nucleotide identity to KHUV (80%), followed by ARAV (79%), EBLV-2 (79%), Australia bat lyssavirus (77%), EBLV-1 (77%), Irkut virus (76%), Shimoni virus (76%), RABV (73-75%) and Duvenhage virus (75%). Lagos bat virus (72-74%), Mokola virus (72%), and West Caucasian bat virus CBV (72%) showed the highest divergence to BBLV. Also, phylogenetic analysis based on concatenated N-P-M-G-L nucleotide sequences showed that BBLV is most closely related to KHUV, followed by EBLV-2 (Figure 2).

## Conclusions

We report the discovery of a lyssavirus (designated as BBLV) from a Natterer’s bat that died with rabies-like clinical signs. Initially, a distinctive pattern in the reaction with a panel of antinucleocapsid monoclonal antibodies indicated the presence of an antigenically atypical isolate. The differentiation from other lyssavirus species was confirmed by phylogenetic analysis (Figure 2).

BBLV is pathogenic because it caused a fatal disease in the Natterer’s bat that was similar to the clinical picture of rabies seen in other bats. Viral antigen was present in many locations of the brain (Figure 1) but surprisingly not in the salivary glands.

Since the exact date of infection is unknown, the incubation period can only be estimated as >4 months. Whether the Natterer’s bat is the natural reservoir species of BBLV or whether it was a cross-species spillover remains a subject for further studies. However, closely related lyssavirus species were also isolated from *Myotis* spp. bats (EBLV-2 from *M. daubentonii*, *M. dasycneme*, KHUV from *M. mystacinus*, and ARAV from *M. blythii*) indicating that *Myotis* spp. bats play a key role in lyssavirus epidemiology. If one considers the history of bat rabies in Europe, it seems unlikely that BBLV had spread from a distant origin into central Europe or that the bat itself was translocated over long distances.

The fact that BBLV has been identified only in 1 bat is puzzling, considering the relatively high level of surveillance in Germany. Also, of 63 Natterer’s bats tested during 1999–2010 in a retrospective study, none tested positive for rabies (T. Müller et al., unpub. data). Germany is the only country where several bat species other than serotine bats, i.e., *Pipistrellus nathusii, Pipistrellus pipistrellus*, and *Plecotus auritus*, have been found infected with EBLV-1 during this study (T. Müller et al., unpub. data), and this is the second discovery in recent years of a new lyssavirus species through routine passive bat rabies surveillance. Also, in 2007 a Daubenton’s bat found on the ground was taken to a rehabilitation center, where it died and subsequently tested positive for EBLV-2 (7). In both cases, the person who took care of the animal had completed the full preexposure vaccination, as a required risk-mitigating measure.

An encounter with a BBLV-infected Natterer’s bat could lead to a fatal outcome because bat lyssaviruses have caused several human cases of infection (4). In Europe, species conservation and research require the handling of bats by bat workers. During 2000–2010, a total of 37,140 handlings were recorded for the Natterer’s bat (Bat Marking Centre, Saxon State Office for Environment and Geology, Dresden, Germany), underlining the need for adequate prophylaxis for bat handlers. If one considers the close phylogenetic relationship between BBLV and EBLV-2 humans who receive rabies prophylaxis will likely be protected. However, recent studies of the antigenic relationships of lyssaviruses have shown the difficulty of interpreting antigenic differences by using sequences alone (14). Thus, in vitro and in vivo cross-neutralization and protection studies with current anti-RABV vaccines are urgently required for assessing the public health risk posed by this new lyssavirus.
